# Impulse Control Disorders in Southern Iraqi Patients Medicated With Cabergoline for Prolactinoma

**DOI:** 10.7759/cureus.58516

**Published:** 2024-04-18

**Authors:** Mohammad M Mohammad, Haider A Alidrisi, Abbas A Mansour

**Affiliations:** 1 Medicine, Faiha Specialized Diabetes, Endocrine and Metabolism Center (FDEMC), Basrah, IRQ; 2 Medicine, College of Medicine, University of Basrah, Basrah, IRQ; 3 Diabetes and Endocrinology, Faiha Specialized Diabetes, Endocrine and Metabolism Center (FDEMC), Basrah, IRQ; 4 Diabetes and Endocrinology, College of Medicine, University of Basrah, Basrah, IRQ

**Keywords:** compulsive eating, hypersexual disorder, impulse control disorders, cabergoline, prolactinoma

## Abstract

Background: Among the patient population in Basrah, Iraq, prolactinoma is the most commonly found pituitary tumor. Impulse control disorders (ICDs) were reportedly associated with these patients being treated with cabergoline. This study aimed to assess the prevalence of ICDs in cabergoline-treated prolactinoma patients versus healthy, matched controls.

Methods: This cross-sectional case-control study was conducted at the Faiha Specialized Diabetes, Endocrine and Metabolism Center (FDEMC) in Basrah, southern Iraq, from January 2023 to May 2023. It included 30 cabergoline-treated prolactinoma patients and 30 healthy, matched controls. The questionnaire for ICDs in Parkinson’s disease was used as a screening tool. Following this, positively screened patients were evaluated using validated criteria accordingly to diagnose impulse control disorders.

Results: The ICDs were diagnosed in nine (30%) cabergoline-treated prolactinoma patients versus two (6.7%) in control (p = 0.02). The most frequent ICD types were hypersexuality and binge eating, while no patient reported pathological gambling. Three patients reported multiple types of ICDs. The patients’ sociodemographic characteristics, prolactinoma duration and size, and cabergoline dose did not correlate significantly with ICD diagnosis.

Conclusions: Treatment with cabergoline is associated with the development of ICDs. Therefore, clinicians should be aware of this disabling side effect to ensure its early detection and treatment.

## Introduction

About 40% to 50% of all pituitary adenomas are prolactinomas, which are a significant contributor to hypogonadism and infertility [1,2]. The mass effect of loss of visual fields is the most concerning symptom [3]. Pituitary adenomas are major pituitary disorders in the patient population of Basrah in southern Iraq, and about 26.9% of these are prolactinomas [4].

Cabergoline is currently the first line of treatment for prolactinoma, with a starting dose of 0.25 mg to 0.5 mg per week [5], which is much lower than that used in patients with Parkinson's disease (up to 3 mg once daily) [6]. Cabergoline has a very long duration of action. Once or twice weekly dosing is typically sufficient for controlling pathological hyperprolactinemia [7], and studies have shown that cabergoline is superior to other dopamine agonists (DA) in terms of efficacy in reducing tumor size and prolactin level [8]. Cabergoline exerts its effect through the activation of D2 and D3 receptors. Activation of D3 receptors in the brain may be responsible for the development of abnormal behaviors in patients developing impulse control disorders (ICDs), as shown by Ahlskog in 2011 [9]. Interest in ICDs is on the rise, particularly in patients with Parkinson's disease receiving dopamine replacement medication, and this suspicion was raised by Molina et al. in 2000 when they noticed pathological gambling among patients receiving DA therapy for Parkinson's disease [10].

Impulse control disorders include pathological gambling, compulsive shopping, excessive eating, and hypersexuality. According to a recent study, ICDs are linked to conditions including fibromyalgia, progressive supranuclear palsy, multiple system atrophy, restless legs syndrome, and multiple system atrophy that are also treated with dopaminergic medicines (dopamine agonists) [11]. These disorders are defined by excessive and/or hazardous desires and behaviors that seriously affect social and occupational functioning as well as generate legal and financial issues [12].

Studies involving a large population and indeed randomized control trials for the incidence of ICDs in patients being treated with cabergoline for prolactinoma are lacking, and this issue is largely underestimated due to a lack of awareness especially in our society due to social limitations. This study aimed to assess the prevalence of ICDs in cabergoline-treated prolactinoma patients versus healthy, matched controls.

## Materials and methods

This cross-sectional case-control study was carried out at the Faiha Specialized Diabetes, Endocrine and Metabolism Center (FDEMC) in Basrah, Iraq, from January to May 2023. It was approved by the Ethics Committee of FDEMC (approval no. 12/23/23). Patients included in the study consented to be enrolled with a written form. The study was approved based on the ethical standards of the FDEMC Research Committee and ethical approval was given

Patients diagnosed with prolactinoma and on cabergoline treatment for at least six months were included in the study. Age, gender, and BMI-matched controls were included for comparison. Patients with a treatment course lasting less than six months, patients with a history of previous psychiatric disorders, patients on other types of treatment with adverse psychiatric effects, patients with known organ dysfunction impairing adequate mental function, and patients with mental disabilities were excluded from the study.

Diagnosis of ICDs

All cases and controls were screened first using the validated questionnaire for ICDs in Parkinson’s disease (QUIP). This questionnaire is composed of four parts and has been previously used for studies of ICDs in Parkinson's disease and restless leg syndrome. Its validity was studied in 2009 [13].

The screening for our study was done using the QUIP in a direct interview. To avoid gender bias, questions regarding sexual activity in female patients were asked by a female doctor. For those who were screened positive by QUIP, a second evaluation using the Diagnostic and Statistical Manual of Mental Disorders (DSM) questionnaires was conducted to confirm the diagnosis of ICDs according to valid criteria for each type of ICD. Patients who were positively screened for hypersexual disorder were evaluated using the proposed criteria for hypersexual disorder according to DSM V [14]. Those who screened positive for compulsive eating were also evaluated using the DSM V criteria application [15].

Compulsive buying disorder was diagnosed per the diagnostic criteria from the 2021 Delphi consensus study after screening positive by QUIP [16]. No patient was screened positive for pathological gambling, mostly due to the unavailability of gambling in our society. Other domains (repeated cleaning, and door closing) were also diagnosed using the DSM V criteria.

Statistical analysis

The SPSS Statistics version 26 (IBM Corp., Armonk, NY, USA) was used for data analysis, with a p-value < 0.05 being significant. The quantitative and qualitative variables were summarized as mean ± standard deviation and numbers (%), respectively. The correlations between qualitative variables were done using the chi-square test and the Fisher exact test. And the correlations between qualitative and quantitative variables were done using the independent Student's t-test.

## Results

The cases and control groups were matched regarding sociodemographic characteristics, as shown in Table 1. Patients with prolactinoma were on cabergoline for a mean duration of 2.9 ± 2.3 years and a current cabergoline dose of 0.6 ± 0.3 mg. Seventeen patients had macroadenoma on presentation; the mean prolactin was 1120.4 ± 600.7 ng/ml at presentation and 68.9 ± 76.8 ng/ml currently.

**Table 1:** General characteristics of the study population (n = 60) PRL: Prolactin

Variable	Prolactinoma group (total n = 30): Mean ± SD or n (%)	Controls (total n = 30): Mean ± SD or n (%)	p-value
Age (years)	37.0 ± 11.8	33.2 ± 10.6	0.2
Age at diagnosis (years)	34.1 ± 11.6		
BMI (kg/m2)	32.4 ± 6.3	30.3 ± 2.9	0.1
Men	14 (46.7)	18 (60.0)	0.3
Women	16 (53.3)	12 (40.0)	
Marital status			
Married	26 (86.7)	23 (76.7)	0.2
Unmarried	3 (10)	7 (23.3)	
Divorced	1 (3.3)	0	
Occupation			
Employed	7 (23.3)	10 (33.3)	0.4
Unemployed	23 (76.7)	20 (66.7)	
Education			
Primary	13 (43.4)	8 (26.7)	0.4
Secondary	7 (23.3)	10 (33.3)	
College	10 (33.3)	12 (40)	
Microadenoma	13 (43.3)		
Macroadenoma	17 (56.7)		
Duration of treatment (years)	2.9 ± 2.3		
Baseline PRL (ng/ml) (normal range: male 4-23 ng/mL and female 4-30 ng/mL)	1120.4 ± 600.7		
Current PRL (ng/ml)	68.9 ± 76.8		
Cabergoline dose (mg) per week			
Maximum	0.8 ± 0.4		
Current	0.6 ± 0.3		

**Figure 1:**
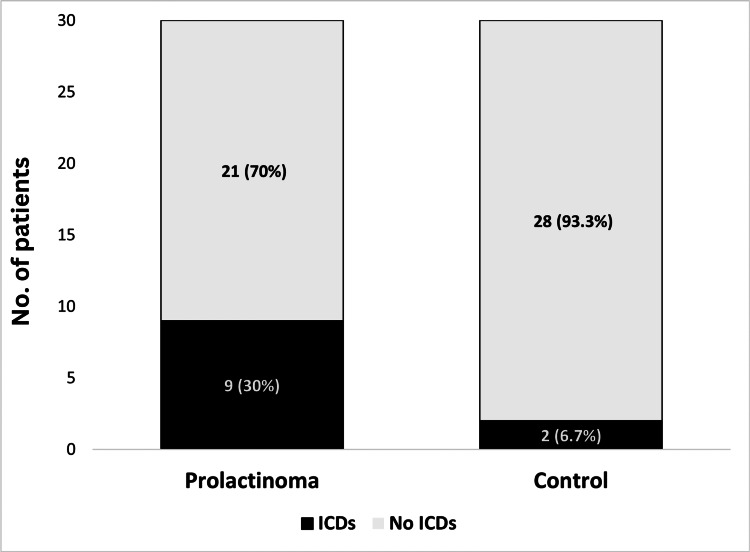
Prevalence of ICDs among patients with prolactinoma versus controls Odds ratio and 95% confidence interval 6 (1.1-30.7), p = 0.02 per the chi-square test. ICDs: Impulse control disorders

Impulse control disorders were diagnosed in nine patients within the case group (30%), and only two patients were diagnosed with ICDs within the control group (p = 0.02), as shown in Figure 1. 

**Figure 2:**
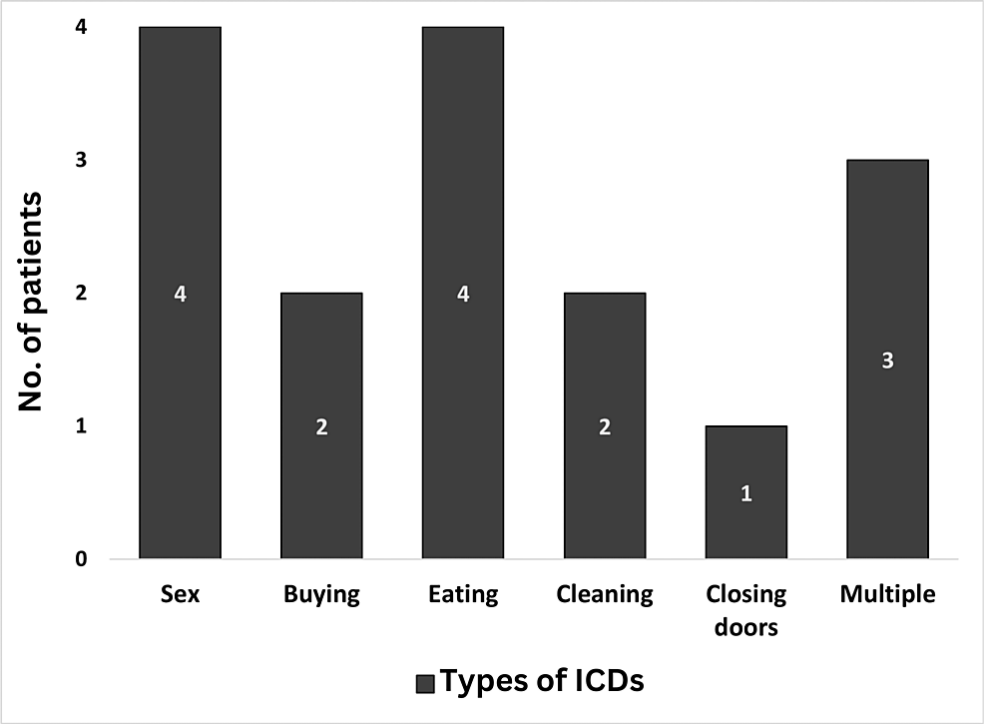
Frequencies of different types of ICDs ICDs: Impulse control disorders

Figure 2 shows the frequencies of different types of ICDs in the case group. Four patients had hypersexuality (only men affected), four patients had compulsive eating, two patients had compulsive shopping, two patients had repeated cleaning, and one patient had repeated door closing. Three patients had ICDs with multiple ICD types.

The patients' sociodemographic characteristics, prolactinoma duration, adenoma size, and cabergoline dose did not correlate significantly with the ICD diagnosis. However, the patients with prolactinoma affected by ICDs had higher maximum cabergoline doses during their treatment course (1.0 ± 0.5 mg versus 0.6 ± 0.2 mg), but with no statistical difference (Table 2).

**Table 2:** Variables and their effects on ICDs among patients with prolactinoma £: Chi-square p-value, α: Fisher exact p-value, µ: Independent Student's t-test p-value ICDs: Impulse control disorders, PRL: Prolactin

Variable	ICDs (total n = 9): Mean ± SD or n (%)	No ICDs (total n = 21): Mean ± SD or n (%)	p-value ^£^
Age (years)	34.7 ± 9.3	38.0 ± 12.2	0.4
Age at diagnosis (years)	32.1 ± 9.9	35.0 ± 12.4	0.5
BMI (kg/m2)	32.3 ± 7.5	32.4 ± 5.9	0.9
Men	5 (35.7)	9 (64.3)	0.6 ^α^
Women	4 (25)	12 (75)	
Marital status			
Married	7 (26.9)	19 (73.1)	0.4^ α^
Unmarried	2 (66.7)	1 (33.3)	
Divorced	0	1 (100)	
Occupation			
Employed	1 (14.3)	6 (85.7)	0.3
Unemployed	8 (34.8)	15 (65.2)	
Education			
Primary	5 (38.5)	8 (61.5)	0.7^ α^
Secondary	2 (28.6)	5 (71.4)	
College	2 (20)	8 (80)	
Microadenoma	4 (30.8)	9 (69.2)	0.6^ α^
Macroadenoma	5 (29.4)	12 (70.6)	
Duration of treatment (years)	2.7 ± 1.8	2.9 ± 2.6	0.7
Baseline PRL (ng/ml) (range: male 4-23 ng/mL and female 4-30 ng/mL)	309.6 ± 171.9	717.2 ± 1312.6	0.3 ^µ^
Current PRL (ng/ml)	88.7 ± 74.6	60.4 ± 78.0	0.3^ µ^
Cabergoline dose (mg) per week			
Maximum	1.0 ± 0.5	0.6 ± 0.2	0.07
Current	0.8 ± 0.5	0.5 ± 0.2	0.2

## Discussion

This is the first study assessing the prevalence of ICDs in patients taking cabergoline for the treatment of prolactinoma in Iraq. It shows a prevalence of ICDs in 30% of cabergoline-treated prolactinoma patients. The most common type of ICD observed in this study was compulsive buying and hypersexuality, while no cases of gambling were detected, probably due to the prohibition of gambling in Iraq. Interestingly, some of the patients reported symptoms of extreme nervousness, irritability, and being easily provoked shortly after starting cabergoline therapy. This suggests that some types of ICDs may not be detected by QUIP, or there may be early symptoms that do not meet the criteria for the diagnosis of ICDs. De Sousa et al. in 2020 suggested the addition of other types of impulsive activities like excessive caffeine intake, exercise, and video games, and this is consistent with our findings [17].

In 2019, a multicenter study detected an ICD prevalence of 17%, with hypersexuality being the most common type, occurring mostly in males, as in our study [18]. Another study found a prevalence of 25.8% in the cabergoline-treated group as compared to 15% in the non-cabergoline-treated group, and this did not reach statistical significance, but compared to our study, the prevalence of ICDs in the control group was much higher [19]. The study by Bancos et al. had comparable results, with 24.8% in patients versus 17.1% in the control group, but was non-significant [20]. Overall, there is a large variation in the prevalence of ICDs in different studies (ranging from 0% to 60%) [21]. This large difference may suggest social and economic factors affecting the occurrence and even the type of ICDs [22]. Another explanation is attributed to the fact that most studies use self-administered questionnaires or online questionnaires and not direct surveys by the doctors themselves. On the other hand, some studies used different methods for diagnosing ICDs and different types of questionnaires.

There are studies suggesting that genetics may affect the susceptibility to ICDs in patients on cabergoline; for example, a study found that the prevalence of the DRD3 p.Ser9Gly (rs6280) CT genotype in Indian patients with Parkinsonism is a risk factor for developing ICDs [23]. Another study found that polymorphisms in the number of genes may increase the incidence of ICDs in cabergoline-treated prolactinoma significantly [24].

In general, the effect of gender on the prevalence of ICDs was not significant, but hypersexuality occurred only in men, and this may be attributed to societal norms in addition to reporting bias by female patients despite using a female doctor to evaluate the patients. This was the same as reported by a study in the USA in 2019, as most hypersexuality occurred in male patients [17]. The effect of other variables like dose or duration of treatment on the prevalence of ICDs was not significant, but the dose of cabergoline was numerically higher in cases versus controls. De Sousa et al. found the resolution of symptoms after cabergoline dose reduction [25], indicating that developing ICDs is dose-dependent. And though the dose used for the treatment of prolactinoma is very low compared to Parkinson's disease, ICDs were still seen in patients treated with cabergoline for both diseases.

So, what should we do if ICDs occur? This may be one of the most important questions to answer after raising this association. There are several options in this regard. Sometimes ICDs could resolve spontaneously, but this may lead to severe social consequences [26]. Reducing to the lowest possible dose may be an option since it was effective in some case reports [11,27]. Furthermore, sertraline was effective for the treatment of ICDs, so using selective serotonin reuptake inhibitors may be an option [27]. Surgical management of prolactinoma could be an option for patients with severe symptoms [28].

Despite being based on a small sample size, these results raise the question of clinicians’ awareness about the occurrence of ICDs in patients treated with cabergoline and the need for further large cohort studies. This study has some limitations. First, it is a single-center study with a small sample size. Thus, a larger, multicenter cohort study would be advisable to assess this relationship between cabergoline and ICDs. Second, due to population norms and prohibitions, it was difficult to interview females, especially regarding hypersexuality, despite being queried by female doctors. Third, patients in our society prefer not to consult psychiatrists for mental disorders due to shyness, and this may affect the exact prevalence of ICDs in patients with previously undiagnosed mental disorders.

## Conclusions

Impulse control disorders were prevalent in 30% of patients with prolactinoma on cabergoline treatment. The most frequent ICD types were hypersexuality and binge eating, and some patients reported multiple types of ICDs. The patients’ socio-demographic characteristics, prolactinoma duration and size, and cabergoline dose did not correlate significantly with ICD diagnosis. Clinicians should be aware of this significant side effect, detect it early, and prevent its negative impacts. Further, larger studies are recommended to detect cofounders and other behavioral side effects not included in QUIP.
